# Kidney Stones as Minerals: How Methods from Geology Could Inform Urolithiasis Treatment

**DOI:** 10.3390/jcm14030997

**Published:** 2025-02-05

**Authors:** Elena Popova, Sergey Tkachev, Artur Shapoval, Anastasia Karpenko, Yuliya Lee, Pavel Chislov, Boris Ershov, Danila Golub, Gevorg Galechyan, Daniil Bogoedov, Anastasiya Akovantseva, Elvira Gafarova, Roman Musaelyan, Maria Schekleina, Stuart Clark, Stanislav Ali, Alim Dymov, Andrey Vinarov, Petr Glybochko, Peter Timashev

**Affiliations:** 1 Federal Research and Clinical Center of Specialized Medical Care and Medical Technologies, Moscow 115682, Russia; elenapopova.prm@gmail.com; 2 Institute for Regenerative Medicine, Sechenov University, Moscow 119991, Russia; 3 School of Minerals and Energy Resources Engineering, University of New South Wales, Sydney, NSW 2052, Australia; 4 Institute for Urology and Reproductive Health, Sechenov University, Moscow 119991, Russia; 5 GridPoint Dynamics LLC, Moscow 123060, Russia; 6 N.N. Semenov Federal Research Center for Chemical Physics, Russian Academy of Sciences, Moscow 119334, Russia; 7 Dokuchaev Soil Science Institute, Moscow 109017, Russia; 8 Department of Petrology and Volcanology, Moscow State University, Moscow 119991, Russia

**Keywords:** kidney stones, urolithiasis, laser lithotripsy, stone analysis, basic endourological research, stone recurrence prevention

## Abstract

Despite the recent advances in minimally invasive surgery, kidney stones still pose a significant clinical challenge due to their high recurrence rate of 50% in 5–10 years after the first stone episode. Using the methods of geosciences and biology, the GeoBioMed approach treats kidney stones as biogenic minerals, offering a novel perspective on their formation and dissolution processes. In this review, we discuss kidney stones’ structural and mechanical properties as emerging biomarkers of urolithiasis, emphasizing the importance of a comprehensive stone analysis in developing personalized treatment strategies. By focusing on unexplored properties like crystalline architecture, porosity, permeability, cleavage, and fracture, alongside the conventionally used composition and morphology, we show how these stone characteristics influence the treatment efficacy and the disease recurrence. This review also highlights the potential of advanced imaging techniques to uncover novel biomarkers, contributing to a deeper understanding of stone pathogenesis. We discuss how the interdisciplinary collaboration within the GeoBioMed approach aims to enhance the diagnostic accuracy, improve the treatment outcomes, and reduce the recurrence of urolithiasis.

## 1. Introduction

Urinary stone disease (USD) is a very common condition that affects up to 14.8% of the population worldwide [1]. Epidemiological studies show that the overall prevalence and the incidence of USD have been increasing over the last several decades [2,3,4,5]. Despite the development of minimally invasive surgery for USD treatment, kidney stones remain a largely recurrent disease with a relapse rate of 50% in 5–10 years after the first stone episode [6,7,8]. Such a high recurrence rate makes the search for novel prognostic biomarkers [9], inhibitors of stone growth [10,11], and stone removal techniques [12,13,14] essential lines of research.

To address the challenge of recurrent urolithiasis, the GeoBioMed approach, which considers kidney stones as biogenic minerals, has been introduced, combining viewpoints from geology, urology, and biology [15,16]. The GeoBioMed framework, first proposed in 2019 [17], assumes that the formation of urinary stones can be understood by studying the universal processes of biomineralization and diagenetic phase transitions, which include the stepwise events of crystal precipitation and dissolution [16]. During diagenetic phase transitions, a stone undergoes physical, chemical, and biochemical changes [16]. In contrast to the conventional approaches for the investigation and classification of kidney stones, which assume that stones usually are composed of one or several minerals, the GeoBioMed approach emphasizes that kidney stones should be evaluated in the context of the sequence of diagenetic phase transitions, which could be applicable to all types of kidney stones, regardless of their composition or place of genesis in the kidney [16]. For example, spherules of amorphous calcium phosphate and hydroxyapatite coalesce to form crystals with calcium oxalate dehydrate crystals and concentric zoning, which indicate nonequilibrium precipitation from the supersaturated urine [16]. By finding a way to interrupt one of the stages of the diagenetic transition, we could investigate new therapeutic agents to prevent recurrence of USD.

However, the conventional methods of morphological or spectral analysis, such as Fourier-transform infrared spectroscopy and Raman spectroscopy, cannot detect small amounts of the crystalline phase and nanolayers of organic matrix proteins that are involved in diagenesis or evaluate the internal structure of the sample [18]. Additionally, by holistically examining not only the morphology of a stone but also the stone’s layering and fracture patterns, crystalline architecture, and porosity, researchers may decipher a record of the patient’s metabolic state throughout the duration of stone formation. Thus, thin sections of the stone could play the role of biopsy tissue slides for diagnostic processes [16].

Therefore, researchers can apply the fundamental principles and methods from geosciences and biology such as thin-section analysis of minerals [19] or transcriptomics [20] in order to investigate USD pathogenesis, possible treatments, and novel prognostic biomarkers. The techniques that the GeoBioMed approach uses include micro-CT as well as advanced specialized techniques of microscopy such as polarized light, super-resolution autofluorescence, and scanning electron microscopy [16].

Like rocks and minerals, kidney stones can be characterized by their structural and mechanical properties, obtained by means of a wide range of methods from geosciences. The composition and morphology of kidney stones have been studied extensively [21]. These parameters were shown to play a crucial role in the diagnosis and development of personalized treatment for USD [18,22]. Other properties, including crystalline architecture, porosity, permeability, cleavage, and fracture, have been less thoroughly studied to date and could serve as new prognostic markers of USD. Furthermore, porosity and permeability might influence the effectiveness of dissolution therapy, while cleavage and fracture might affect lithotripsy [23,24,25,26].

**Figure 1:**
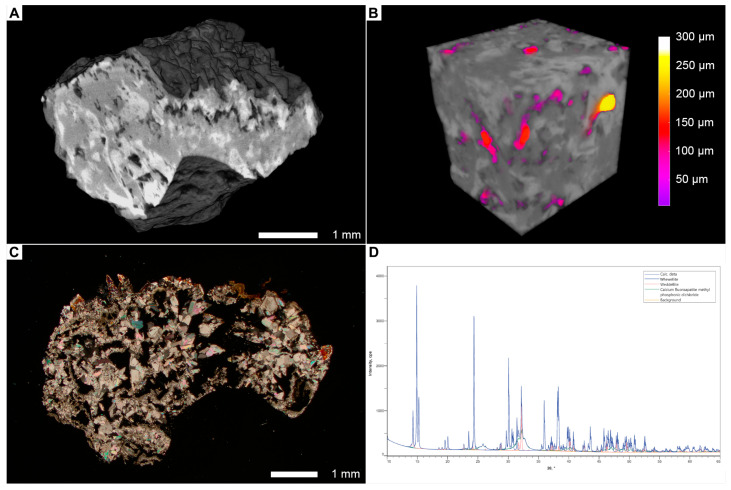
Multimodal analysis of a stone specimen within the GeoBioMed paradigm. (**A**). Computed microtomography (micro-CT) image of a mixed kidney stone composed of calcium fluorapatite, calcium oxalate monohydrate (COM), and calcium oxalate dihydrate (COD). (**B**). Three-dimensional visualization of the internal pore network with color representations of pore diameters. (**C**). Thin-section analysis of a stone showing a layered structure and a complex mineral composition. (**D**). X-ray diffraction analysis enabling precise determination of mineral component proportions.

In this review, we focus on these properties and their investigation methods. As mentioned above, the multimodal analysis of stones within the GeoBioMed paradigm uses various methods, such as computed microtomography(micro-CT), polarized light microscopy, and X-ray diffraction analysis (Figure 1, details see in Supplementary Materials). It provides a complex characterization of stone properties, which can assist in improving the characterization of stone morphology, investigation of new treatments for dissolution therapy, achieving stone-free status, and decreasing the risk of recurrence. Furthermore, based on the vast amount of collected patient data and the properties of stones, we can contribute to developing clinical decision support systems.

## 2. Search Strategy

We searched the PubMed, Cochrane library, and Google Scholar databases for the following terms: “kidney stone disease”, “urolithiasis”, “recurrent stone disease”, “proteomics”, “radiomics”, “morphology”, “crystalline architecture”, “porosity”, “permeability”, “hardness”, “cleavage”, “fracture”, “residual stone fragments’, “stone-free rate”, “GeoBioMed”, and “geobiology”. Then, dedicated search strings were created based on a combination of the aforementioned keywords without any restrictions on the time frame of publication. As the next step, we filtered the articles according to the inclusion and exclusion criteria listed below:

The inclusion criteria were

- Full-length original articles and reviews;
- Articles by authors highly influential in the field.

The exclusion criteria were

- Similar, duplicate, or not very relevant studies;
- Literature published in languages other than English.

Upon checking their type and content, we included studies in this review. Additionally, we identified other articles through reference lists that satisfied the criteria mentioned above and subsequently considered them in our review. We performed a narrative synthesis to analyze the studies.

## 3. Stone Morphology and Crystalline Architecture

Descriptions of stone morphology and crystalline architecture include various parameters, such as the size, shape, color, textural characteristics of the surface and section, crystal habits, and forms [18]. The spatial scale of these parameters ranges from nanometers to centimeters [18,21,25]. Some tests, such as light stereomicroscopy, are usually performed by physicians at the point of care in pathology or laboratory medicine departments. Others, such as scanning electron microscopy (SEM), polarized light microscopy (PLM), and computed microtomography(micro-CT), are carried out at research institutions, since these methods require specialized laboratory equipment. The existing evidence shows that morphological analysis is crucial from both the fundamental and practical perspectives because it aids diagnosing [18,22,27], understanding the stone formation processes [28] and initially investigating the molecules that inhibit crystal growth [10,29].

In order to determine the etiology of urolithiasis, the current guidelines [30,31,32] recommend analytical techniques, such as X-ray powder diffraction (XRD) and Fourier-transform infrared spectroscopy (FTIR), after stone passage or surgery. However, the reliance on these methods alone can lead to misdiagnosis, especially in the cases of rare stone types (e.g., 2,8-dihydroxyadenine or drug-induced calculi) or the presence of different crystalline phases in the stone [21,33]. As an example of misdiagnosis, Bazin et al. [34] reported that the crystalline conversion of calcium oxalate dihydrate (COD, weddellite) to calcium oxalate monohydrate (COM, whewellite) introduces a contradiction: FTIR spectra indicate the presence of COM (induced by hyperoxaluria), while one could observe bipyramid crystallites, a morphological feature specific to COD (related to hypercalciuria), via polarized light microscopy, micro-CT, and SEM. Thus, the analysis of a stone’s crystalline architecture can accurately direct the physician toward particular pathological conditions, such as genetic metabolic disorders, like absorptive hypercalciuria type 1, renal tubular acidosis, and primary hyperoxaluria type 1, which may rapidly lead to end-stage renal failure [18]. It should be noted that the results of morphological and crystalline architecture studies must always be supported by a 24 h urinalysis and, in the case of suggested genetic metabolic disorders, by genetic testing [18,31].

### 3.1. Light Stereomicroscopy

Examining a kidney stone using a light stereomicroscope is a less expensive and more accessible method compared to chemical assays via FTIR or XRD. Optical imaging of stone morphology can identify a variety of pathological conditions, including cystinuria, enteric hyperoxaluria, and urinary tract infection (UTI) [22]. The Daudon morpho-constitutional classification is based on the morphological features of kidney stones, supported by the information about the predominant chemical substances, which are revealed via physical methods (XRD, FTIR, etc.) [18,22,35]. The morpho-constitutional classification includes six groups of stones composed of calcium oxalate monohydrate or calcium oxalate dihydrate, uric acid, urate salts, carbapatite, struvite, brushite, and proteins, which are subdivided into morphological subtypes corresponding to specific conditions [18,21]. Despite the advantages discussed above, the visual determination of morphology typically depends on an operator, meaning it requires certain experience in the identification of various mineral and nonmineral compounds, with substantial experience required in cases involving rarely seen stone types. Another limitation is the inaccurate identification of mixed stone types that could contain different components in the crystalline and amorphous phases, since the external appearance of the stone type may not be representative [36]. It is also noteworthy that comorbidities such as type 2 diabetes mellitus or UTI cause additional lithogenic processes, which might affect the morphology of the previously formed stones or unpredictably alter the repeated recrystallization and dissolution during stone growth [22].

To address these challenges, researchers should develop systems for stone identification and assessing crystalline architecture based on deep learning methods, using the images obtained from a light stereomicrosope, as this approach has been already applied for digital cameras [37], smartphones [38], and endoscopic camera systems [39,40,41]. We suggest it would be beneficial in order to enhance the diagnostic accuracy obtained through morpho-constitutional analysis. However, aforementioned systems can only identify a single mineral that occupies the largest proportion in stone images. Since kidney stones are usually mixed [18] and the composition of the outer surface differs from that of the inner surface of the stone [36], one might apply multilabel image classification, which is widely used in geosciences for mineral identification. In the nature, minerals also exist in the associated form, which makes identification impossible with traditional machine learning algorithms [42,43]. In multilabel image classification, each image contains multiple objects to be recognized, and these objects are related [42]. For example, Wu et al. reported that a multilabel classification model achieved a mean average precision of 85.26% and located the identified minerals based on a 183,688-image dataset of 36 common minerals [42]. However, we found recent studies in which the multilabel image classification was successfully implemented for images obtained with dual-energy computed tomography [44] and an endoscopic camera system [45], which makes this approach also applicable for images obtained via a light stereomicroscope.

### 3.2. Petrographic Analysis

**Figure 2:**
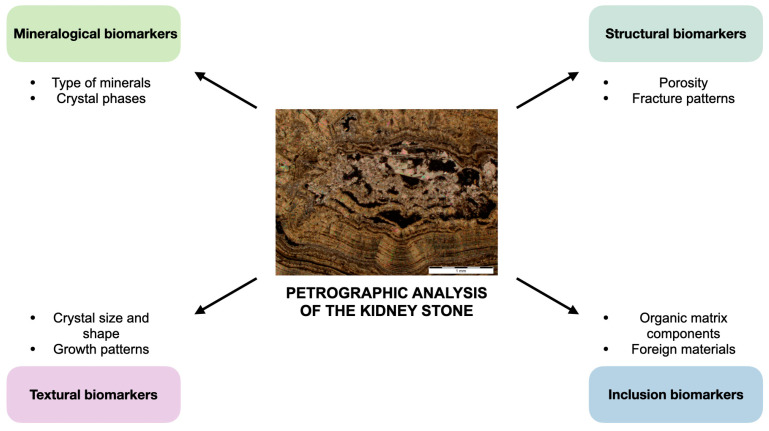
Petrographic analysis of thin sections reveals history of kidney function. Petrographic analysis allows identification of many new biomarkers. In addition to the precise determination of the type of kidney stone, its stage of development, the pattern of mineralogy, crystalline architecture, stratigraphy, and diagenesis, a kidney stone can serve as a historical record of the function of the kidney and the organism as a whole. Thus, using petrographic analysis, it is possible to determine the metabolic function of the patient’s body and better understand the patient’s condition in order to treat associated disorders.

Petrographic analysis (or thin-section analysis) is a technique for studying the optical properties and textures of minerals in thin slices. Sample preparation includes cutting a small thin slice (20–30 μm) of a stone and mounting it on a glass slide. The slice is then grounded and polished to a thin, transparent section that can be examined using polarized light, phase contrast, and confocal autofluorescence (CAF) or super-resolution auto-fluorescence microscopy (SRAF) [19,46]. For example, Figure 1C and Figure 2 represent an image of the thin section of a kidney stone obtained via polarized light microscopy (PLM). A subsequent analysis of this section showed the presence of COM and COD crystallites, which were organized into layers.

Guidelines on USD from the European Association of Urology recommend PLM for kidney stone analysis [32]. Different minerals exhibit different optical properties, such as pleochroism, i.e., color variations that follow crystal orientation, and extinction angles, i.e., angles at which minerals appear dark. By observing these optical properties under polarized light, one can identify the mineral composition of a sample. The optical properties of a material limit PLM’s applicability, since this method cannot distinguish between the amorphous phase and smaller amounts of the crystalline phase in COM. Since PLM is an operator-dependent technique, it is hard to standardize [47]. In geosciences, similar limitations to PLM were overcome by employing AI-assisted techniques [48] that helped to identify pyroclastic rocks [49] and Eocene carbonates [50]. To date, there are no known applications of AI to thin-section images of kidney stones.

Sivaguru et al. proposed a new classification scheme for kidney stones following the results of the thin-section analysis that revealed the diagenetic phase transitions in CaOx stones [46] (Table 1) [16]. This classification challenged the conventional point of view on the composition and formation of kidney stones. In 2018, in the seminal work by Sivaguru et al., the thin-section analysis performed via CAF and SRAF showed that CaOx kidney stones repeatedly dissolved in vivo, as suggested earlier by Bazin et al. [34,46]. The authors thoroughly characterized the historical sequence of events during crystal growth, which included concentric zonations of free-floating COM and COD, formation of the COM nanolayering cortex, and dissolution and recrystallisation of the COM cortex [46]. In 2023, Todorov et al. were the first to observe the crystal growth structure via the SRAF and CAF microscopy of shock-wave lithotripsy (SWL) particles in thin sections [19]. These particles are undetectable via clinical computed tomography (CT) and become seed points for crystallization, thereby contributing to the high rates of stone recurrence after SWL. Currently, there are no published papers about the thin-section analysis of the crystalline architecture and diagenetic phase transitions in other stone types, about the thin-section analysis of particles produced by laser lithotripters (thulium fiber, pulsed thulium:YAG, and holmium:YAG lasers), or their estimation on the Wentworth grain size scale. Such comparative studies may shed light on the causes of relapses after laser lithotripsy, especially after reaching a stone-free state, defined as the absence of any residual stones on radiologic evaluation performed 4 weeks postoperatively, since the size of residual particles is much smaller than the resolution of clinical CT.

Since each layer could represent a distinct episode of growth, as the rings of a tree trunk indicate growth periods, these layering patterns visualized via petrographic analysis could provide a chronological record of the stone’s formation and reflect changes in the metabolic processes and urinary composition over time [16]. Variations in the layer thickness and crystalline architecture could reveal changes in the urinary saturation of stone-forming minerals, changes in the urine pH, or the presence of inhibitors or promoters of crystallization [16]. For example, struvite-layered stone could indicate periods of urinary tract infection [52], since these layers are often more porous, and they reflect the rapid stone growth associated with infection [53]. By studying the composition, sequence, and characteristics of the layers, we could analyze the history of stone formation, estimate the duration of stone growth, and identify periods of increasing urolithiasis activity.

### 3.3. Micro-CT

Computed microtomography (micro-CT) is a rapidly evolving nondestructive imaging technique widely applied in fields such as materials science, biomedical research, and geosciences [54,55,56]. It facilitates the in-depth examination of the internal and external structures of various samples by generating high-resolution three-dimensional images or cross-sectional slices, with the resolution reaching 2 µm or finer. Micro-CT functions by rotating a specimen and capturing a sequence of X-ray images from diverse angles. These 2D X-ray images are subsequently reconstructed to produce a comprehensive 3D volume, enabling further analysis and visualization. Figure 1A–C represent an image analysis workflow. For example, Figure 1A shows the internal structure of the stone, which was composed of calcium fluorapatite, COM and COD, and the composition was determined via X-ray diffraction analysis (Figure 1D). Image analysis included binarization and segmentation. Binarization is a conversion of a color or grayscale image into a two-color black and white image (Figure 1A). Segmentation identifies individual objects in the image, e.g., pores of the stone or stone material (Figure 1B).

Micro-CT has been utilized since 2001 to analyze various properties of kidney stones and has been covered in several detailed and comprehensive reviews. For example, the reviews by Williams et al. in 2010 [28] and 2022 [57] as well as the one by Borofsky et al. in 2016 [58] highlight the role of micro-CT imaging as a tool for the high-resolution distinction of stone components; identification of the regions of interest for analysis using other methods, e.g., thin-section analysis [19]; and determination of the 3D structure of stones for a better understanding of the etiology of USD (Figure 1A). For instance, micro-CT 3D surface renderings reveal the features related to specific anatomical phenotypes, such as Randall’s plaque phenotype, in which concavities with the presence of apatite (revealed by lower X-ray attenuation) and luminal spaces at the point of contact with the kidney papillary tissues are observed [28].

Williams and colleagues emphasized that micro-CT alone cannot be applied to determine stone type using morphological characteristics. Instead, such properties could be defined using light stereomicroscopy. However, when combined with FTIR, micro-CT may notably enhance the accuracy of the latter [57,58]. Stones consisting of organic materials (e.g., urates, 1-methyl uric acid, and 2,8-dihydroxyadenine) were reported to have an X-ray attenuation value similar to that of uric acid without special morphological features on micro-CT [57]. The combination of X-ray attenuation measurements and gross examination of the stone sample was rather effective: specimens that yielded X-ray attenuation in the middle range (7000–12,000) could be identified by their visual appearance.

In the geosciences, micro-CT is an established tool for examining rock microstructures. It provides data on three-dimensional internal morphology, aiding in studying fluid flow in porous samples [59,60,61]. Micro-CT is also used for fracture analysis, detailing fracture networks, morphology, distribution, and connectivity, which are essential for studies of fluid migration in fractured systems [62,63,64,65]. Micro-CT aids with grain size and shape analysis in sedimentary geology, allowing for the assessment of grain distributions and inferences on depositional environments and diagenetic processes. The capability of micro-CT to differentiate minerals through X-ray attenuation is used to segment the mineral phases in rock samples (a technique also known as rock typing), thus facilitating mineralogical analysis [66]. When combined with machine learning, micro-CT can automate mineral identification, fracture propagation prediction, and mineral properties estimation [67]. This integration of micro-CT data analysis with machine learning methods from geosciences can enhance the effectiveness of the GeoBioMed approach applied to kidney stones by improving mineralogical analysis, elucidating formation processes, and predicting stone properties [17,19,46].

### 3.4. Scanning Electron Microscopy

Since the 1980s, SEM has been a common method for the structural characterization of kidney stones [68,69]. In 2021, Bazin et al. extensively reviewed its role in the examination of kidney stones and highlighted that SEM alone could not be a routine diagnostic tool due to the diversity of crystal morphology and, more importantly, due to the existing phase transitions of COD to COM and brushite to apatite [70]. However, in the case of primary hyperoxaluria, COM has specific morphology, and crystallites are present in spherical structures resembling balls of wool [71]. In the case of hypercalciuria, the diagenetic transitions of COD to COM make an accurate diagnosis impossible, since FTIR indicates the presence of amorphous COM, while SEM and micro-CT observations reveal bipyramidal morphology with large structural defects that indicate the existence of a phase transition [34]. Additionally, density-dependent color-scanning electron microscopy (DDC-SEM) can be applied to examine the morphology of crystals in kidney tissues, e.g., showing that the specific morphology of COM crystallites in biopsies indicates primary hyperoxaluria [72].

In the geosciences, mineral mapping allows identifying the distribution of different compounds in a sample or a region of interest via SEM combined with energy-dispersive X-ray spectrometry (EDS) [73,74,75]. Elemental analysis using SEM imaging with EDS was shown to be applicable to morpho-compositional studies [76]. However, mineral mapping has not been used so far to characterize the spatial distribution of the mineral phases in kidney stones. Automated mineralogy and petrography systems detect and quantify small amounts (<1%) of minerals in samples as well as generate digital images of the analyzed samples on a pixel-by-pixel, particle-by-particle basis. Hence, mineral mapping by combining morphological analysis and chemical assays may reveal the role of trace heavy elements as seed points for coalescence and excessive crystallization, e.g., gadolinium from contrast agents [77] as well as lead and cadmium from contaminated air and water [78], since the presence of these pollutants significantly increases the incidence of USD [79,80]. Furthermore, SEM-EDS mineral mapping may reveal permineralization around damaged renal epithelial and bacterial cells, which leads to the formation of alternating layers with various mineral phases that contain distinct concentrations of salts, organic matter, or specific ions [81]. For instance, mineral mapping of the Mari Ermi Sardinian stromatolites showed that the permineralization of the bacteria sheaths leads to the enrichment of calcite with magnesium and the formation of brighter laminae in the stromatolitic layers [82].

## 4. Proteins of the Stone Matrix

The molecular mechanisms of stone growth still remain unknown. The cellular and molecular niches of human renal cells differ between patients with calcium oxalate (CaOx) stone disease and healthy individuals. The levels of MMP7 and MMP9 are significantly elevated in the urine of patients with CaOx stone disease, and their levels correlate with disease activity. For instance, Canela et al. found a correlation between the expression of metalloproteinases MMP7 and MMP9 [20]. Since patients with idiopathic urolithiasis may have no abnormal blood biochemical parameters [83], stones and their components, including proteins, could serve as historical records of kidney function and fluid homeostasis, aiding in the diagnosis [16]. Therefore, many studies have been dedicated to the proteomics of kidney stones [84,85]. Among the at least 1400 proteins that can be found in stones [84], some may be essential for kidney stone formation and could be unexplored targets for new therapies for recurrent USD. The most common stone matrix proteins include S100A8, S100A9, uromodulin, albumin, and osteopontin [84]. However, the spatial distribution of proteins can be a significant factor affecting the stone formation processes. For example, Tanaka et al. revealed that osteopontin (OPN) and renal prothrombin fragment 1 (RPTF-1) were distributed inside COM and COD crystals, while calgranulin A was distributed outside the crystals [86]. The distribution of OPN and RPTF-1 in COM and COD crystals is homogeneous, having a mosaic texture and periodic distribution parallel to certain crystal faces.

## 5. Porosity and Permeability

**Figure 3:**
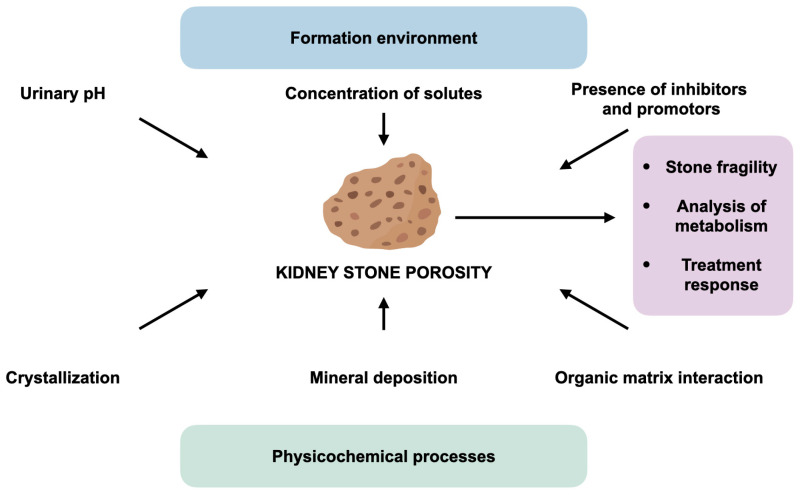
Porosity and permeability. Porosity may play a key role in the renal stone formation, since it facilitates the penetration of a supersaturated solution, as well as contributes to the removal of some mineral components during the stone development. Porosity may also indicate the heterogeneous ability of different types of stones to succumb to the action of lithotripsy and, therefore, may serve as a biomarker for the selection of adequate crushing regimes. Furthermore, sufficient porosity in certain types of renal stones may allow new drug agents based on mineralogical principles to penetrate the stone and facilitate its destruction or directed transformation into more amenable structures. In addition, the porosity of kidney stones may allow bacteria to colonize kidney stones from within, thus creating the prerequisites for the infectious complication of urolithiasis.

Porosity is the percentage of voids in a stone that contain a supersaturated aqueous solution of urine [46,87]. Like rocks and minerals, kidney stones initially have inherent primary porosity [88] that can be altered by diagenetic phase transitions, comorbidities, deformations during SWL and laser lithotripsy, and medication intake. These processes are hypothesized to produce the secondary porosity of kidney stones; therefore, the porosity could serve as a biomarker for revealing the causes of pore formation (Figure 3). For instance, micro-CT and thin-section analysis revealed the presence of moldic porosity, which is a type of secondary porosity created through the dissolution of a pre-existing constituent of a stone [16]. Changes in the pore size distribution and pore geometry could increase the reactive surface area of particles produced during SWL or laser lithotripsy, which may lead to the recurrence of kidney stones and ultimately reduce the efficiency of interventions.

### 5.1. Porosity and Outcomes of Lithotripsy

Pores are usually observed in all types of kidney stones, especially in mixed stones, i.e., those composed of two or more types of minerals, where different mineral phases are in contact. Manzoor et al. [25] observed that, among the stone types, the lowest porosity was found in pure stones (0.23% in COM, 4.36% in uric acid, and 7.90% in struvite), and the highest porosity was observed in mixed COM–COD stones (from 2.44% in struvite-apatite, 2.88% in mixed COM–uric acid, 7.53% in COM–COD–apatite, and up to 10.77% in COM–COD). The authors hypothesized that the porosity variation among the mixed stones may pose a challenge during SWL because highly porous stones, such as COD and struvite, are more susceptible to SWL than less porous stones, such as COM [25].

Giannossi et al. noted the misconception that porous structures correspond to softer kidney stones, confusing the hardness of each mineral with the hardness of its aggregates [23]. The authors refuted that misconception by observing that in the case of most calcium phosphates, despite a hardness of give on the Mohs scale, phosphate kidney stones are among the easiest ones to be destroyed using lithotripsy techniques. However, there is some evidence that questions the effect of porosity on the effectiveness of lithotripsy. Cavalli et al. [89] found no correlation between the porosity in COM, struvite, apatite, uric acid, cystine, and whitlockite (i.e., a type of calcium phosphate stones) samples and their post-SWL fragmentation rate. In another study, with micro-CT alone used to evaluate the internal structure in brushite calculi, no correlation was observed between the fragility of the calculi and the evaluated internal structures [90]. It should be noted that the influence of stone porosity on stone-free status has not yet been investigated.

### 5.2. Porosity–Permeability Relationships

There are two types of porosity: effective (or open) porosity from pores that are interconnected and permit the flow of fluids through them and ineffective (or closed) porosity from pore spaces that trap fluids [88]. Consequently, effective porosity influences permeability, which is defined as a measure of the ability of a stone to transmit fluids. In the case of kidney stones, a higher effective porosity increases permeability and may lead to faster stone growth or bacterial colonization. Manzoor et al. [52] found a distinct variation in the porosity at different stages of crystal growth in the presence and absence of bacteria, namely, *Enterobacter cloacae* and *Pseudomonas aeruginosa*. The cross-sectional architecture of struvite in the presence of bacteria was rougher and more porous compared to the control samples [52].

Furthermore, porosity and permeability can influence the effectiveness of dissolution therapy [26]. Dissolution and precipitation reactions are potentially able to alter porosity and permeability [91]. While porosity generally increases with mineral dissolution and decreases with precipitation, the corresponding permeability alterations are complex, where the predictive capabilities remain limited [91]. Julià et al. reported that the most-porous uric acid stones dissolved with theobromine treatment at a higher rate than the most-compact stones [26]. To investigate new therapeutics for oral chemolysis, one needs to model the flow of a supersaturated aqueous solution of the urine inside the pore network of stones.

To assess the relationships between the porosity and susceptibility to lithotripsy and dissolution therapy, it is necessary to quantitatively describe the pore structure of kidney stones [88]. Direct methods of porosity measurement include water saturation, gas expansion and adsorption, and mercury intrusion porosimetry [88]. All of these methods only measure the effective (connected) porosity of the samples [88]. Direct methods are necessary to verify and enhance the precision of imaging techniques in porosity studies. For instance, mercury intrusion porosimetry (MIP), which has never been used in studies for the porosity measurement of kidney stones, is a technique that saturates a sample with mercury under increasing pressure and measures the volume of mercury that permeates the pore space. By correlating the pressure needed for mercury intrusion into the pore size distribution, both the total and specific pore volumes can be determined [60,92,93,94]. While MIP can investigate nanopores and thus characterize the porosity more precisely than micro-CT, the latter provides a spatial description of the pore structure, despite the limitations in resolution (micrometer resolution compared to the nanometer resolution of MIP) [95]. Before MIP, one should evaluate the internal structure of the stone by performing an analysis of its pore network using micro-CT, since an open pore is needed for mercury intrusion.

Pore segmentation and porosity analysis have become two of the most important applications of micro-CT in geosciences [96,97,98]. These techniques refer to the fluid flow modeling used to study the flow of fluids through a porous network [99,100]. This area of research seems to be closely related to the complex urodynamic studies of fluid mechanical modeling in the renal pelvis [86,87] and stented ureter [101,102,103]. Porosity analysis involves acquiring hundreds or even thousands of X-ray projection images at 360 degrees around a sample, which are then used to reconstruct the 3D volume of the sample. For example, Figure 1B shows a three-dimensional visualization of a stone’s internal pore network and represents the superimposed volume of the stone, with the pore network showing the actual distribution of pores inside the stone material. The 3D volume can be used to create a pore-scale simulation model that predicts the fluid flow properties, e.g., permeability, based on the pore geometry and fluid properties. The open-source GeoChemFoam tool can model complex flow processes, including multiphase transport with interface transfer, single-phase flow in multiscale porous media, and reactive transport with mineral dissolution. One may apply this aforementioned fluid flow modeling to study the dynamics of a supersaturated aqueous solution of the urine in kidney stones using the data on the stones’ porosity obtained via micro-CT [104].

## 6. Residual Stone Fragments

The mechanical properties of kidney stones include their ability to resist deformation and the cracks’ progression to complete failure [105]. Kidney stones break at very low deformation, behaving as a brittle material, like the minerals of which they are composed [106]. However, it should be noted that the mechanical properties of kidney stones, which are composed of mineral components and proteins, may differ significantly from those of mineral species. The mechanical properties require further investigation since they may affect the results of extracorporeal ultrasonic SWL and laser lithotripsy techniques [106].

According to the reviewed literature, the mechanical properties of kidney stones and the ways they could affect the short- and long-term outcomes of stone removal techniques have not been investigated. Currently, the clinical data on the long-term outcomes of laser lithotripsy are also limited. It remains unclear how the type of laser and the methods of stone destruction (i.e., dusting or fragmentation with extraction) affect the risk of recurrence. There are still disputes around the long-term outcomes of holmium:YAG lasers compared to thulium fiber, superpulsed thulium fiber, and pulsed solid-state thulium:YAG ones. Hence, we suggest that prospective trials with long-term follow-up (>2 years) on patients, including a detailed investigation of the kidney stone properties (e.g., micro-CT to examine the morphology and porosity, AFM to measure the surface roughness of stone fragments, and thin-section analysis to study the cleavage and diagenetic processes), would be beneficial. Furthermore, we assume that samples derived from patients with a history of recurrent USD are highly desirable in order to collect data on the properties of recurrent kidney stones.

### 6.1. Does the Hardness of a Stone Really Matter?

The mechanical properties of minerals and rocks reflecting their response to deformation may not fully explain the results of lithotripsy techniques. For instance, hardness, which has been studied via the Vickers hardness test [107] and depth sensing indentation [108], does not seem to be relevant in the context of SWL and laser lithotripsy, since hardness characterizes the ability of a mineral to resist the impact of a harder material [109]. Perhaps of much greater importance are thermal conductivity and heat capacity, since laser energy is converted into heat (i.e., photothermal effect) and causes physical phenomena that destroy a stone, which include cavitation (i.e., superheated vapor bubbles’ collapse resulting in pressure drops that destroy the stone’s substance), ablation (i.e., vaporization of the stone substance), formation of internal stress and cracking of the stone due to the slow heat release, and shock wave formation due to rapid heat release in the small volume of a stone [12,110]. The photothermal effect, occurring in the dusting mode of laser lithotripsy, changes the crystalline architecture of the stone, e.g., resulting in the conversion of the carbapatite crystalline phase to its amorphous phase, COD to COM, and brushite to hydroxyapatite [27].

### 6.2. Cleavage and Fracture

During fragmentation, a stone is broken into several pieces, which are then extracted. However, there is a risk of the retropulsion of relatively large stone fragments, which may lead to obstruction or recurrence. Large fragments (2–4 mm) are more likely to cause recrystallization after fragmentation since the total external surface area has been shown to be a first-order approximation of the reactive surface area in both PCNL-derived fragments and SWL-derived particles [19]. In this case, a thulium fiber laser is superior to a holmium:YAG one because the former provides dusting with a finer particle size in a shorter time [111]. The small particles, less than 1–2 mm in diameter [112], derived after dusting can be easily removed with water flow. Studies have emphasized the importance of complete stone clearance since the rates of repeated surgical interventions are higher in patients with clinically insignificant residual fragments (<4 mm) than in those in stone-free groups [113,114,115]. These fragments might promote excessive recrystallization, which leads to stone regrowth and recurrence. Innovative techniques, such as those using pulsed-thulium:YAG or superpulsed thulium fiber lasers, aid in achieving complete stone-free status since they produce a fine dust containing particle sizes ≤ 250 µm, which can easily pass down the ureter [116,117,118]. However, it remains unclear how such small residual particles might affect the long-term outcomes of laser lithotripsy. Furthermore, the interpretation of the existing evidence might be complicated due to the variability in laser techniques and their settings, the differences in the follow-up time, and the application of various stone-free rate definitions [119].

The reactive surface areas of laser-produced residual particles may substantially contribute to the recurrence of urolithiasis, as shown for SWL-derived particles [19]. During extracorporeal SWL, a clinician must strike a subtle balance between fragmenting the stone into small nonobstructive pieces, protecting renal tissues from the negative effects of cavitation (i.e., a mechanism through which vapor bubbles in a fluid grow and collapse as a result of fluctuations in the local pressure) [120,121], and achieving the complete removal of stones.

Since calcium oxalate stones can break along their planes of weakness during SWL [122] (this property of minerals is known as cleavage [123]), these undetectable residual fragments can facilitate epitaxy, a type of crystal growth in which new crystalline layers are formed with one or more well-defined orientations with respect to the crystalline seed layer [124]. In turn, fracture refers to the way a mineral breaks in the absence of specific planes [105]. Unlike cleavage, which occurs along the planes of weakness of atoms, fractures are uneven or rough-surfaced, resulting from the breaking of the mineral’s internal bonds [105]. After such a fracture, the original mineral may break up into smaller fractions [105]. These rough reactive surface areas can have a significant impact on the crystallization rate [19]. The surface roughness increases the effective surface area and therefore increases the substrate-generated supersaturation with respect to crystal nucleation [19]. The surface area of crystals is a factor in the rate coefficient, which can impact the crystallization kinetics [125]. The reactive surface area and surface roughness of the residual particles of destroyed stones can be quantitatively measured via atomic force microscopy (AFM) [126], and these properties are closely related to morphology. AFM has been successfully applied in morphological investigations of crystals [127] and stones [128], as well as for imaging molecular step growth and testing growth-inhibiting molecules in situ [10,29,129,130,131,132,133]. However, the types of destruction among various groups of stones, the reactive surface area, and the surface roughness of laser-lithotripsy-derived particles have not been investigated.

## 7. Future Perspectives

### 7.1. Metabolic Biomarkers

**Figure 4:**
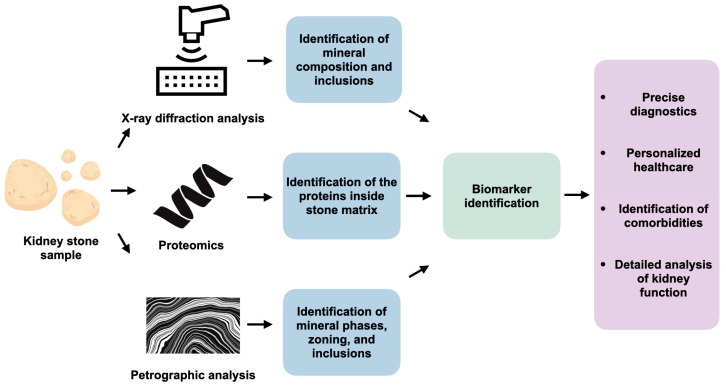
Novel methods for investigating biomarkers of urolithiasis.

Since USD is a systemic disorder, the high recurrence rates after surgical stone removal increase the cost burden of the disease. Furthermore, recurrence significantly reduces the quality of life of patients who are forced to seek medical care again. However, since kidney stones can be considered a historical record of the kidneys’ function, new diagnostic approaches are opening up opportunities for detailed metabolic studies in search of the cause of the disease. Petrographic analysis and XRD, which have been proven to be reliable mineralogical methods of investigation, may become key tools for finding new biomarkers within the structure of kidney stones. Combined with proteomic analysis, they make it possible to search for new biomarkers responsible for the development of urolithiasis, to implement personalized treatment, and to identify comorbidities based on a detailed understanding of the metabolism and renal function of a particular patient (Figure 4).

### 7.2. Integrating Radiomics with the GeoBioMed Approach

Radiomics can be used to predict stone fragility to the choose optimal settings in laser lithotripsy, thereby being crucial for the effective management of urolithiasis. Single-energy computed tomography (CT), dual-energy CT (DECT), and radiomic analysis facilitate the classification of stone type and the acquisition of essential data concerning their responsiveness to various treatment approaches [134]. For example, Ferrero et al. analyzed internal features such as the surface roughness, CT number ratio, and co-occurrence matrices [135]; Wang et al. utilized three-dimensional computed tomography texture analysis [136]. Lidén et al. combined single-energy CT with a three-dimensional Laplacian image filter for the prediction of the stone’s chemical composition [137]. Hokamp et al. integrated dual-energy CT with machine learning to predict the main component of pure and mixed kidney stones ex vivo [138]. Moreover, the identification of infection-related stones was advanced through the work by Zheng et al. [139], who also considered clinical factors like the presence of urease-producing bacteria in the urine and the urine’s pH using multivariate logistic regression analysis. Zou et al. integrated clinical variables and radiomic features to develop a machine learning model, and the logistic regression showed accuracies of 78.1% in predicting the stone-free rate after PCNL. Zwanenburg et al. highlighted the necessity of the standardization and calibration of radiomic features, especially in multicentric studies [140]. By integrating the obtained micro-CT and clinical CT data with deep and machine learning algorithms, radiomics analysis could accurately predict stone composition and fragility. Further research is needed to establish the correlations between the mineralogical properties of urinary stones, their structural and chemical composition, and clinical data.

### 7.3. Investigating the Optimal Settings of Laser Lithotripsy

**Figure 5:**
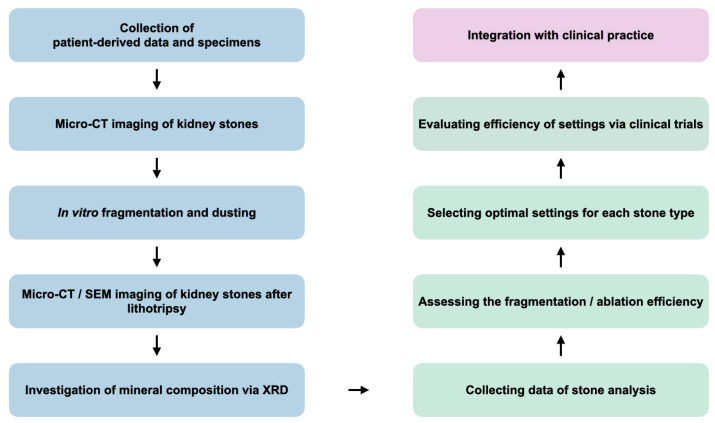
Experimental workflow for selecting the optimal settings for each type of kidney stone. micro-CT, computed microtomography; SEM, scanning electron microcopy, XRD, X-ray diffraction.

In order to effectively model lithotripsy, it is necessary to utilize the full range of fracture analysis and crack propagation examination applied in the geosciences and use micro-CT, chemical analysis (e.g., XRD), and SEM for this purpose. A proposed experimental approach includes several steps (Figure 5). Initially, patients are enrolled, with their data and specimens collected. Subsequently, in vitro lithotripsy testing with different modes is performed under controlled conditions. The lithotripsy-exposed stones are then visualized again using micro-CT to determine how the fracture has spread within the stone and what structural changes have been observed there. Additionally, SEM can be applied to visualize the changes at a high resolution. XRD is then used to determine the mineral composition of the stone, and all the data obtained are integrated with the patient data including CT scans. In this way, it is possible to investigate how different lithotripsy regimens affect the internal structure of a stone depending on its chemical composition and to find out whether it is possible to predict a stone’s ability to break up on the basis of CT scans. 

### 7.4. Stone Properties as Fingerprints for Novel Classifications and Clinical Decision Support Systems

The current classifications of kidney stones, presented in Table 1, along with the advantages and disadvantages of each scheme, typically consider only one or two properties. Among those considered are the composition, morphology, diagenetic phase transitions during stone formation, and anatomical location. However, state-of-the-art techniques such as machine learning have shown promise in identifying novel patterns and relationships in large sets of data, e.g., electronic health records [141]. Based on these extended sets of data, which include a patient’s clinical history, as well as a full description of the stones, integrative classification schemes combine the advantages of multiple existing classification methods to direct physicians toward the required therapeutic options and to predict relapses of USD. Furthermore, there are additional benefits of collecting data on stone properties; for example, these properties can be used to conduct in silico modeling of stone formation and dissolution by simulating the interactions with growth-inhibiting molecules [10].

## 8. Limitations of This Review

As mentioned above, there are already several reviews on the techniques and methods of kidney stone investigation. However, to date, this paper is the first narrative review in which both the structural and mechanical properties of kidney stones and the methods of their investigation are covered. Our review has some limitations. The literature was not systematically reviewed, the quality of the included studies was not assessed, nor was a meta-analysis of the literature conducted. Since our review is not systematic, some studies may not have been considered.

## 9. Conclusions

**Figure 6:**
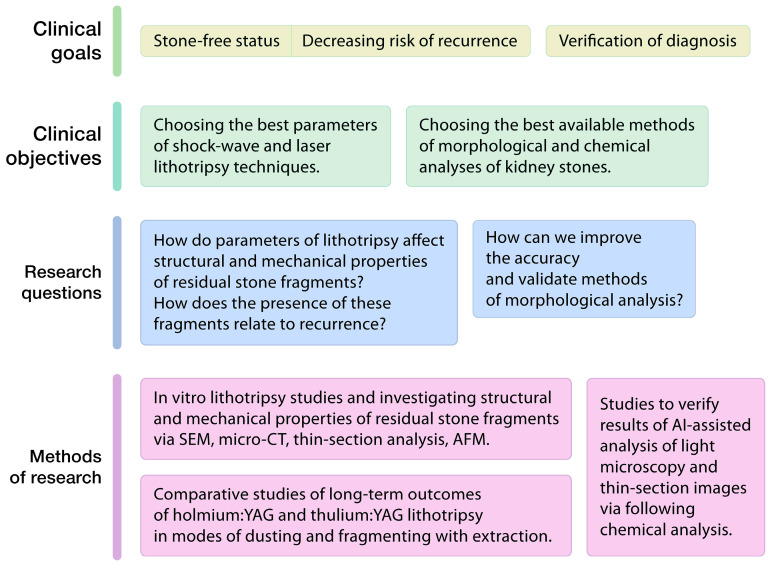
Clinical goals that could be achieved through research in the context of the GeoBioMed approach. SEM, scanning electron microscopy; micro-CT, computed microtomography; AFM, atomic force microscopy; AI, artificial intelligence; holmium:YAG, holmium:yttrium-aluminium-garnet; thulium:YAG, thulium:yttrium-aluminium-garnet.

In this review, we discussed the properties of kidney stones and the methods of their analysis. We chose the crystalline architecture, proteins of the stone matrix, porosity, permeability, hardness, cleavage, and fracture based on the conventional classification system for rock and mineral properties in geoscience. Some of these properties, especially morphology, have been extensively studied and have considerable significance in clinical practice. In Figure 6, we highlight some clinical goals, such as stone-free status, decreasing the risk of recurrence, and the verification of diagnosis, which could be achieved through research in the context of the GeoBioMed approach.

The approaches derived from geosciences, such as the use of multilabel image classification to standardize the analysis of thin-section images obtained via stereomicroscopy and polarized light microscopy, could improve diagnostic accuracy. Certain properties, such as porosity and cleavage, require further investigation. The mechanical strength of kidney stones describes their resistance to fragmentation, which may have implications for their growth and response to treatments such as lithotripsy. It is necessary to estimate how laser lithotripsy affects fracture propagation and the size of the particles after fragmentation, as it was shown for SWL-derived particles. Understanding the mechanical features enables researchers to fabricate artificial kidney stones. Furthermore, the porosity of kidney stones may have an impact on the formation of bacterial biofilms and infectious stones and influence the results of dissolution therapy.

It would be beneficial if patients with recurrent USD could be included in longitudinal studies with the analysis of all available kidney stone properties using the techniques reviewed above. We hypothesize that this could facilitate the modeling of USD etiology and kidney stone formation and thus be of assistance in the selection of the best treatment options. The growth in patient datasets makes the use of machine learning a potential method for further identification of the links between patient characteristics, treatments, and outcomes. These results could be used to develop clinical decision support systems.

## Figures and Tables

**Table 1:** Current systems of urinary stone classification.

Classification Scheme Based on	Classifiers	Advantages	Limitations	References
Stone composition	Calcium oxalate Hydroxyapatite Uric acid Struvite Brushite Cystine Mixed	Revealed predominant substances by IR or XRD—relatively inexpensive and typically available methods. Could orient physician toward underlying pathological conditions (UTI, hyperoxaluria). Revealed by combination of FTIR or XRD and optical microscopy—relatively inexpensive and typically available methods. Could orient physician toward underlying pathological conditions more accurately.	Fails to distinguish rare stone types (e.g., 2,8-dihydroxyadenine; drug-induced calculi) or presence of different crystalline phases (e.g., in the case of primary hyperoxaluria type 1).	[51]
Morphology and stone composition	Appearance Section Composition	Revealed by combination of FTIR or XRD and optical microscopy—relatively inexpensive and typically available methods. Could orient physician toward underlying pathological conditions more accurately.	Requires advanced skills of stone identification or SEM application. Identification of rare and mixed stone types can be challenging or impossible.	[21]
Diagenetic phase transitions	Precipitation pathways: (1) Direct (SSAS-HAP, SSAS-COD, SSAS-COM); (2) Stepwise (ACP-HAP, HAP-COD, COD-COM, COM-COM); (3) Bypass (PILP-ACP, ACP-COD, ACP-COM, HAP-COM).	Revealed by thin-section analysis via PLM or autofluorescence microscopy—relatively inexpensive methods. Allow for search of growth-inhibiting molecules (target the initial formation of ACP and HAP spherules and prevent aggregation of free-floating COD and COM).	Require advanced skills in mineralogical and petrological analysis. The clinical significance remains to be investigated. To date, developed only for CaOx stones.	[16]
Anatomical location and pathways for kidney stone formation	(1) Stones fixed to the renal papilla at sites of interstitial apatite plaque (Randall’s plaque), as seen in idiopathic calcium oxalate stone formers. (2) Stones attached to plugs protruding from the openings of ducts of Bellini, as seen in hyperoxaluria and distal tubular acidosis. (3) Stones forming in free solution in the renal collection system, as in cystinuria.	Could orient physician toward underlying pathological conditions.	This classification cannot be applied when stones are passed naturally or after SWL treatment. Studies revealed the presence of mineral deposits throughout the entire volume of the renal papilla [16].	[16]

IR, infrared spectroscopy; XRD, X-ray diffraction; UTI, urinary tract infection; SEM, scanning electron microscopy; SSAS, supersaturated aqueous solution of urine; COM, calcium oxalate monohydrate; COD, calcium oxalate dihydrate; HAP, hydroxyapatite; ACP, amorphous calcium phosphate; PILP, polymer-induced liquid precursor; PLM, polarized light microscopy; CaOx, calcium oxalate; SWL, shock-wave lithotripsy.

## Data Availability

Not applicable.

## Supplementary Materials

The following supporting information can be downloaded at: https://www.mdpi.com/article/10.3390/jcm14030997/s1, Supplementary Material S1.
