# Supplementary material for: Kidney Stones as Minerals: How Methods from Geology Could Inform Urolithiasis Treatment
Source: J Clin Med. 2025 Feb 5;14(3):997. doi: 10.3390/jcm14030997 (PMC11818645; doi:10.3390/jcm14030997)
Supplement: Supplementary file 1 [file jcm-14-00997-s001.zip › jcm-3378928-supplementary.pdf]

Kidney stone sample analysis

To illustrate the principle of a multimodal analysis of kidney stones within the GeoBioMed paradigm (Figure 1), we investigated a single kidney stone sample of mixed COM-COD composition using X-ray micro-CT imaging, thin-section microscopy, and X-ray diffraction analysis. The informed consent was obtained from a patient, and the kidney stone was collected after the standard percutaneous nephrolithotomy procedure. X-ray micro-CT imaging was performed using the Bruker Skyscan 1276 machine (Bruker, Belgium). The following parameters were used for micro-CT: voltage of 50 kV, current strength of 200 μA, aluminum filter thickness of 0.5 mm. The samples were imaged using 180 mode of scanning (900 projections) with a voxel resolution of 7 μm. The obtained projections were reconstructed in the NRecon program (Bruker, Belgium) and exported as a sequence of images in 16-bit .tif format. Two-dimensional images were acquired using the 3D Slicer open-source software. Binarization, segmentation, and the subsequent porosity analysis were performed using the Bruker CTAn software. The pore network and the superimposed image of the stone material were 3D visualized using the Bruker CTVox software. Transparent polished thin sections of the kidney stone specimen were analyzed using an OLYMPUS BX53 (Olympus, Japan) polarizing microscope with an OLYMPUS DP72 camera and an OLYMPUS TH4-200 reflected light unit. Mineralogical studies were carried out employing the XRD (X-Ray Diffraction) method using a Rigaku SmartLab SE universal X-ray diffractometer (Rigaku, Japan). Imaging mode: Cu radiation (Toshiba 2.2kW) with a wavelength of 1.54178 Å and using a graphite monochromator, tube voltage 40kV, current 50mA, angular speed of the counter (1D) from 4 2θ °/min, step 0.02°, Bragg-Brentano geometry, sample holder for 10 cuvettes. Sample preparation consisted of rubbing the samples to <0.05μm and applying the undirected preparation to the surface of low-background cuvettes. The diffractometer was controlled by theSmartLab Studio software. The qualitative and quantitative mineralogical data processing packages were included into the program. The calculation of mineral ratios was performed using the Rietveld method (WPPF) and a set of cards with the crystallographic information from the PDF2 database.
